# Evolutionary Dynamics-Aware *De Novo* Design of a Thermostable NS1-Derived Diagnostic Protein for Yellow Fever Virus

**DOI:** 10.1021/acsomega.6c07158

**Published:** 2026-09-04

**Authors:** Junior Olimpio Martins, Patrícia da Silva Antunes, Rodrigo Lopes Sanz Duro, Robert Andreata-Santos, Clara Lacerda de Athayde, Gustavo Cabral-Miranda, Luiz Mário Ramos Janini, Franca Fraternali, Carlos Henrique Bezerra da Cruz, Ricardo Durães-Carvalho

**Affiliations:** † Department of Morphology and Genetics, 58804 Federal University of São Paulo, São Paulo, São Paulo 04023-062, Brazil; ‡ Laboratory of Urban Rodent Virology, 28105 Institut Pasteur de São Paulo, São Paulo, São Paulo 05508-020, Brazil; § Postgraduate Program in Microbiology and Immunology, Federal University of São Paulo, São Paulo, São Paulo 04023-062, Brazil; ∥ Anhembi Morumbi University, São Paulo, São Paulo 04546-001, Brazil; ⊥ Laboratory of Genetics and Molecular Cardiology, Heart Institute, Clinical Hospital, Faculty of Medicine, University of São Paulo, São Paulo, São Paulo 05403-000, Brazil; # Institute of Tropical Medicine, Faculty of Medicine, University of São Paulo, São Paulo, São Paulo 05403-000, Brazil; ∇ Department of Microbiology, Immunology and Parasitology, Federal University of São Paulo, São Paulo, São Paulo 04023-062, Brazil; ○ 4919 University College London, Department of Structural and Molecular Biology, Darwin Building, 604 Gower Street, London WC1E 6BT, U.K.; ◆ Interunit Bioinformatics Graduate Program, Institute of Chemistry, University of São Paulo, São Paulo, São Paulo 05508-000, Brazil

## Abstract

Serological diagnosis of neglected arboviruses remains hindered by extensive intra-genus cross-reactivity, compromising both assay specificity and epidemiological accuracy. In this study, we focused on yellow fever virus (YFV) as a model system to design and validate a generalizable *de novo* protein design framework integrating evolutionary genomics with structural modeling to rationally engineer diagnostic antigens with reduced cross-reactivity and improved cost-effectiveness. Through comprehensive evolutionary mapping over time of the YFV polyprotein, we identified conserved yet discriminatory peptide regions within the NS1 protein and selected one peptide to guide scaffold-based protein design while preserving critical structural constraints. As a complementary strategy to develop antibody alternatives for antigen detection assays, we also implemented an *in silico* RNA aptamer design pipeline to circumvent the time and cost limitations of conventional *in vitro* Systematic Evolution of Ligands by EXponential enrichment (SELEX) technique. Nevertheless, NS1-targeting aptamers demonstrated limited sensitivity and specificity upon experimental validation. In contrast, structure-guided design of the NS1-derived protein (YFV-Scaffold) yielded a thermally stable, highly expressible recombinant protein, which demonstrated sensitivity and specificity comparable to those of commercially available full-length NS1 in in-house ELISA assays. Circular dichroism analysis revealed minimal denaturation up to 94 °C and complete recovery of secondary structure upon cooling, underscoring its remarkable structural resilience. Collectively, our results establish an evolutionary dynamics-aware strategy for target-antigen design, providing a scalable platform for the rational design of robust, low-cost diagnostic proteins applicable to a wide range of viral pathogens.

## Graphical abstract

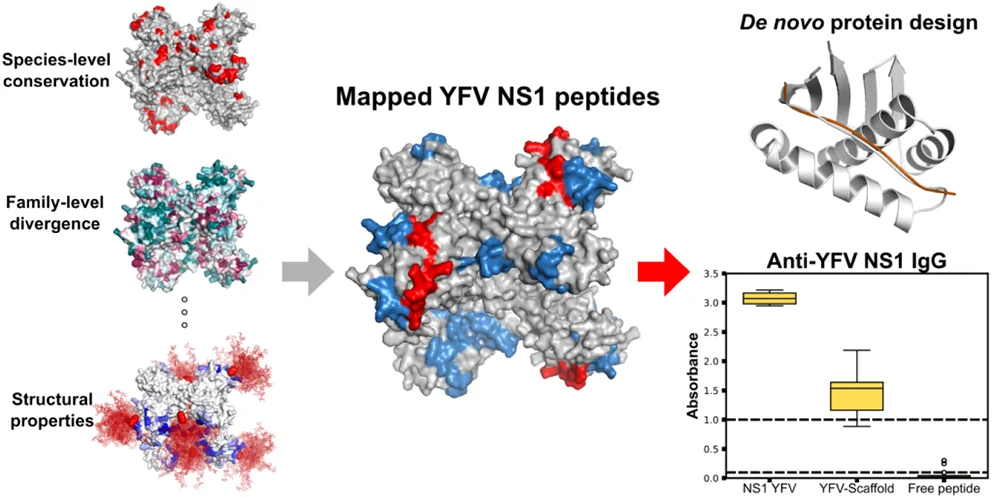

## Introduction

Arboviruses pose major public health challenges in tropical regions due to high mortality, morbidity, and frequent reemergence.^1^ Yellow fever virus (YFV) has long been a pathogen of concern in Latin America because of its severe urban outbreaks. Although eliminated from urban settings, YFV persists in sylvatic cycles involving nonhuman primates.^2^ Despite an effective live attenuated vaccine, outbreaks continue among unvaccinated travelers and vulnerable populations.^3^ Assessing the full public health impact of YFV remains difficult due to three main factors, as follows: clinical similarities to other arboviruses,^4^ a short viremia window for molecular testing,^4^ and high cross-reactivity with other flaviviruses in serological assays.^5,6^ Addressing these limitations requires rapid, specific, and cost-effective diagnostic tools suitable for low-resource settings.

Accurate YFV diagnosis faces several challenges that hinder disease surveillance and patient management. The available diagnostic approaches fall into two categories with distinct drawbacks: molecular (viral genome detection) and serological (antigen or antibody detection) methods.^7^ Among these, real-time PCR (qPCR) is the gold standard for early detection due to its high specificity and sensitivity.^6^ However, its effectiveness is limited by the short viremic period of only a few days after symptom onset,^4^ leading to false-negative results in late-presenting patients.

Conversely, serological methods such as the enzyme-linked immunosorbent assay (ELISA) are essential for detecting late-stage or past infections. Nevertheless, they suffer from substantial false-positive reactivity in regions where dengue (DENV) and Zika (ZIKV) viruses co-circulate.^8^ This low specificity results from both complex patient infection histories and the high degree of genetic and antigenic similarity among flaviviruses, which promotes boosting of cross-reactive antibodies responses during subsequent infections.^8^ To overcome this limitation, several strategies, including both recombinant viral proteins and peptide-based antigen approaches, are under investigation. Recombinant viral proteins closely mimic native antigens, but are cost-prohibitive and technically demanding to produce owing to their large size, complex glycosylation, and preservation of cross-reactive epitopes.^9^ On the other hand, small peptides are more cost-effective and easier to synthesize yet may not replicate native three-dimensional structures, potentially compromising assay sensitivity and specificity.^10,11^

Selecting viral antigens that minimize cross-reactivity while preserving virus-specific recognition is critical for the development of serological diagnostics.^12^ This requires consideration of both sequence and structural properties. For YFV, the nonstructural 1 (NS1) protein offers superior diagnostic potential over the envelope (E) protein, as NS1 is the only secreted viral protein and engages extensively with the immune system.^13,14^ Once the target protein has been selected, antigen regions that are conserved within species yet divergent across related viruses must be identified. In this context, phylogenetic inference provides a robust approach for identifying conserved regions with less sampling bias than consensus sequence-based alignment methods.^15^ However, the identification of suitable peptide targets alone is insufficient, as diagnostic performance also depends on preserving the structural context required for accurate antibody recognition. Accordingly, the selected motifs must be incorporated into protein scaffolds capable of maintaining their native three-dimensional conformations while enabling efficient and scalable production.

Recent advances in artificial intelligence (AI)-driven protein design have created unprecedented opportunities to address this challenge. The advance in AI on *de novo* design of proteins with high atomic accuracy has revolutionized the development of new therapeutic proteins through creative solutions, particularly in the design of vaccinal and diagnostic proteins.^16^ Over the past decades, grafting peptide motifs onto a scaffold protein library via motif-grafting approaches has been the main traditional route to generate functional chimeric proteins for various purposes.^17^ Notwithstanding, the restricted number and diversity of natural proteins and topologies with desirable structural properties, in addition to the complexity of motif-scaffold alignment, have posed significant challenges in engineering such functional constructs.^18^

Following the recent advances in neural networks, boosted by the remarkable success of protein structure prediction algorithms in predicting large and complex folds with unprecedented precision, the engineering of epitope-scaffold proteins has undergone a disruptive transformation, enabling the creation of synthetic and functional proteins never seen in nature by *de novo* design approaches.^16^ Embedding epitopes into novel proteins, meticulously tailored to maintain their native conformations and properties, has become feasible with the emergence of diffusion-based methods capable of creating previously unreported protein topologies with high experimental success rates, without reliance on existing protein libraries.^19,20^ These advancements in neural networks and protein structure predictions have also been translated into the design and structural prediction of nucleic acids, with increasingly accurate applications in aptamer design.^21^ These include algorithms for primary sequence generation, selection and filtering, as well as for tertiary structure prediction of the aptamer alone or in conjunction with a proposed protein binder.^22^ In this context, several immunoreactive and diagnostic proteins have been designed from scratch using similar approaches.^19^

In this work, we designed small epitope-scaffold proteins derived from conserved regions of the YFV NS1 protein for diagnostic applications. Using evolutionary analysis and deep learning-based modeling, we identified multiple highly conserved peptides across distinct YFV lineages. One peptide was selected as the template for *de novo* protein design. The top-ranked candidate was validated *in vitro*, exhibiting favorable structural properties and diagnostic performance comparable to that of full-length NS1, thereby it a promising candidate for YFV-specific diagnostics.

## Results and Discussion

Here, we implemented an evolutionary dynamics-aware *de novo* design strategy integrating phylogenetic conservation mapping, structural epitope characterization, and computational scaffolding to develop a diagnostic protein with high specificity for YFV while minimizing flaviviral cross-reactivity. Bayesian phylodynamics identified NS1 regions conserved over extended evolutionary time scales, with selection analyses revealing episodic rather than pervasive positive selection. A single conserved, surface-exposed NS1 peptide was selected and submitted to *de novo* scaffold design using RFdiffusion and ProteinMPNN for backbone generation and sequence design, yielding a thermostable, highly expressible YFV-Scaffold protein that preserves the native epitope conformation. Experimental validation showed that YFV-Scaffold recapitulates the reactivity profile of full-length NS1 against polyclonal antibodies, whereas the isolated peptide did not, underscoring the essential role of structural scaffolding. In contrast, an *in silico* aptamer design pipeline failed to generate functional binders. Collectively, these results establish evolutionarily informed *de novo* protein design as a scalable route to diagnostic reagents with performance comparable to that of full-length viral antigens.

### YFV Phylodynamics

To identify regions conserved over time and to contextualize the evolutionary history of YFV, we performed a phylodynamics analysis using Bayesian ancestral character reconstruction. This approach allowed us to infer ancestral characters for each site in the polyprotein and estimate the time to the most recent common ancestor (tMRCA), thereby defining the temporal scale over which sequence conservation was assessed. The estimated tMRCA was 1800, with a 95% highest posterior density (HPD) interval ranging from 1599 to 1927 (Figure 1A). This estimate aligns closely with previous YFV phylodynamic studies, which dated the most recent common ancestor of circulating South American lineages to the late 18th or early 19th century.^23,24^ The relatively broad HPD interval reflects the sparse sampling of historical YFV genomes, a limitation common to YFV evolutionary studies.^25^ Our results showed conserved regions encompassing viral diversity accumulated over centuries and provided a robust evolutionary framework that serves as the basis for inferring temporally conserved regions, which were then annotated onto the NS1 protein (Figure 1B). Notably, the temporal conservation mapping revealed that the majority of surface-exposed residues in NS1 have remained invariant since the 19th century, suggesting strong functional or structural constraints that may be exploited for diagnostic targeting.

**Figure 1:**
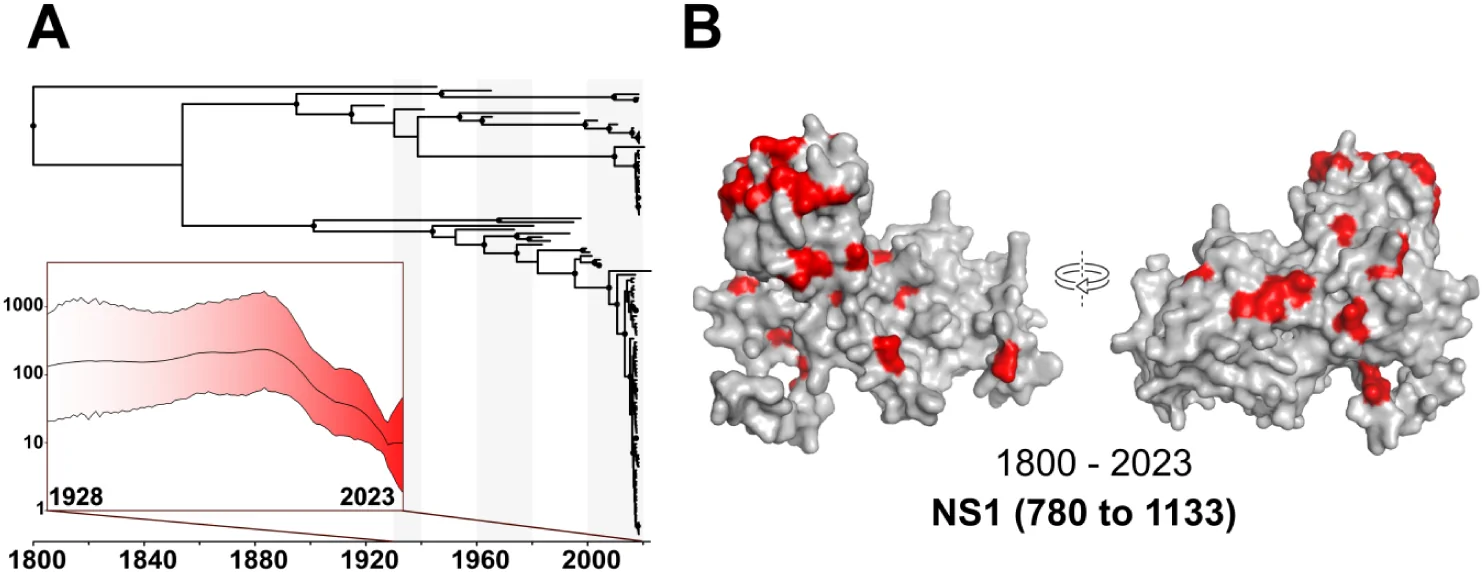
Maximum clade credibility (MCC) trees, population dynamics, and temporal conservation mapping of the YFV NS1 protein. (a) The MCC tree for the YFV polyprotein is shown together with the SkyGrid reconstruction of effective population size over time. The SkyGrid plot (white to light red gradient) displays the estimated median and corresponding 95% HPD interval. Nodes with posterior support ≥80% are indicated by circles. (b) The structure of the NS1 monomer, in which residues inferred to have changed throughout YFV evolutionary history are highlighted in red. NS1 coordinates within the viral polyprotein sequence are shown in parentheses.

Conserved regions were validated using two complementary approaches: reconstruction of ancestral states in the maximum clade credibility (MCC) tree, which captures conservation at the species level, and ConSurf analysis, which reflects conservation at the family level. This dual-level strategy addresses a key limitation of many diagnostic epitope discovery efforts, which often focus solely on interspecies conservation and neglect intraspecies temporal stability.^26^ By requiring both family-level divergence (to minimize cross-reactivity) and species-level temporal conservation (to ensure diagnostic longevity), we aimed to develop diagnostic antigens that are robust to both cross-reactive antibodies and viral evolution. This approach enabled the identification of six distinct conserved regions in NS1, including one region that partially overlapped with adjacent conserved motifs (Figure S1A).

### Selection Analyses

Selection pressure analysis on the NS1 protein revealed limited evidence for pervasive positive selection across the alignment, as no sites under positive selection were identified by SLAC, FEL, or FUBAR under the applied thresholds (p-value ≤ 0.05 for SLAC and FEL, and posterior ≥ 0.9 for FUBAR). This absence of pervasive positive selection is consistent with reports on other flavivirus NS1 proteins, which typically evolve under purifying selection due to their essential roles in viral replication and immune evasion.^27,28^ In contrast, MEME detected three sites under episodic positive selection at a p-value ≤ 0.05 (positions 48, 228, and 307), suggesting that the evolutionary dynamics of this viral protein are dominated by episodes of positive selection in a subset of lineages rather than by divergence along the whole phylogeny, a pattern that can be associated with host immune evasion, functional adaptation, or changes in infection dynamics (e.g., the (re)­emergence of new lineages). Sites 228 and 307 are located within the β-ladder domain of NS1, which has been previously implicated in interactions with complement proteins;^29^ episodic selection at these positions may reflect lineage-specific adaptation to host complement systems.^30^ Site 48 falls within the wing domain, which shares structural similarity to the RIG-I dsRNA response element and may therefore affect NS1-mediated attenuation of dsRNA sensing.^29^ Consistently, none of the positively selected sites overlapped with the conserved regions selected for downstream analyses, indicating that these target regions are evolutionarily stable and less likely to be affected by adaptive sequence variation over time.

### Epitope Characterization, Prediction, and Selection

We used a combination of computational tools to identify potential antigenic regions with high immunological relevance. The analysis incorporated predictions of both linear B-cell epitopes and peptides with high binding affinity to major histocompatibility complex (MHC) class I and class II molecules. This complementary approach was adopted because linear B-cell epitopes are directly involved in antibody recognition, whereas MHC-binding peptides provide information on T-cell immunogenicity (both CD8+ and CD4+) and may help identify regions under broader immune surveillance.^31,32^ Integrating these features enabled the prioritization of conserved NS1 regions with potential relevance for diagnostic antigen design.

Because epitope immunogenicity is strongly influenced by surface accessibility, we next evaluated the potential masking effects of glycan-mediated molecular shields on the native protein. To ensure epitope exposure while avoiding the need to replicate the complex glycosylation patterns of viral surface proteins, we identified and validated O- and N-glycosylation sites using literature data, after which glycans were grafted onto the structures. Glycosylation sites were identified at positions 130 and 208,^33^ enabling the identification of occluded regions on the protein surface (Figure S1B). These glycosylation sites are conserved among flaviviruses and have been shown to modulate NS1 secretion, stability, and immunogenicity.^34^ The presence of glycans at these positions creates a physical shield that may reduce antibody accessibility to nearby epitopes, a phenomenon previously exploited by viruses to evade humoral immunity,^35^ or may themselves act as part of a conformational epitope.^36^ By deliberately selecting epitopes outside these shielded regions, we aimed to maximize diagnostic sensitivity without the need to replicate the complex glycan environment.

Lastly, to select the most promising candidate among the six conserved regions identified, the regions were filtered according to their properties in the native structure, such as secondary structure, lack of glycan shielding, solvent exposure, and epitope proximity (Figure S2). Additionally, regions containing positively selected sites were excluded. This stringent filtering reduced the candidate pool to a single region, a 17-residue motif located in the wing domain of NS1.

### Protein Design and Characterization

A total of 80,000 backbone scaffolds were generated using RFdiffusion, yielding substantial structural diversity, with the proportion of residues classified as α-helices ranging from 0 to 70% across the generated backbones (Figure S3). This broad distribution of secondary structure content reflects RFdiffusion’s ability to sample diverse protein topologies. Such flexibility represents a major advancement over traditional motif-grafting approaches, which are inherently constrained by the diversity of available scaffold libraries and often require extensive manual optimization and remain biased toward known protein folds.^37^

Additionally, while useful as a predictor of design success, full-atom structure prediction is computationally expensive, taking approximately 20 min per structure using ColabFold^38^ or approximately 1 min using AlphaFold3, which is subject to a daily limit on the number of available predictions. Thus, to reduce computational cost and runtime, two backbone properties, number of backbone contacts (NC) and radius of gyration (RG), were selected as filters before reverse folding and full-atom structure prediction, acting as proxies for compactness and stability. This filtering strategy is analogous to the pre-filtering approaches used in high-throughput computational design^16^ and reduced the number of structures requiring expensive full-atom prediction by >99.9%, enabling the evaluation of a large backbone scaffold library with modest computational resources.

While sequence length was not used as a filtering criterion, plotting RG versus NC for each backbone revealed an overall funnel-shaped pattern and an inverse relationship between RG and sequence length (Figure 2A), indicating that the generation process did not introduce an obvious bias toward a specific scaffold architecture. Nonetheless, this pattern did not fully reflect which scaffolds would be selected by the filtering criteria; ultimately, 26 structures passed the RG and NC filters, with lengths ranging from 77 to 120 residues. The funnel-shaped distribution suggests that RG and NC capture complementary aspects of structural quality, with low RG (compactness) and high NC (core formation) jointly predicting designability.^39,40^

**Figure 2:**
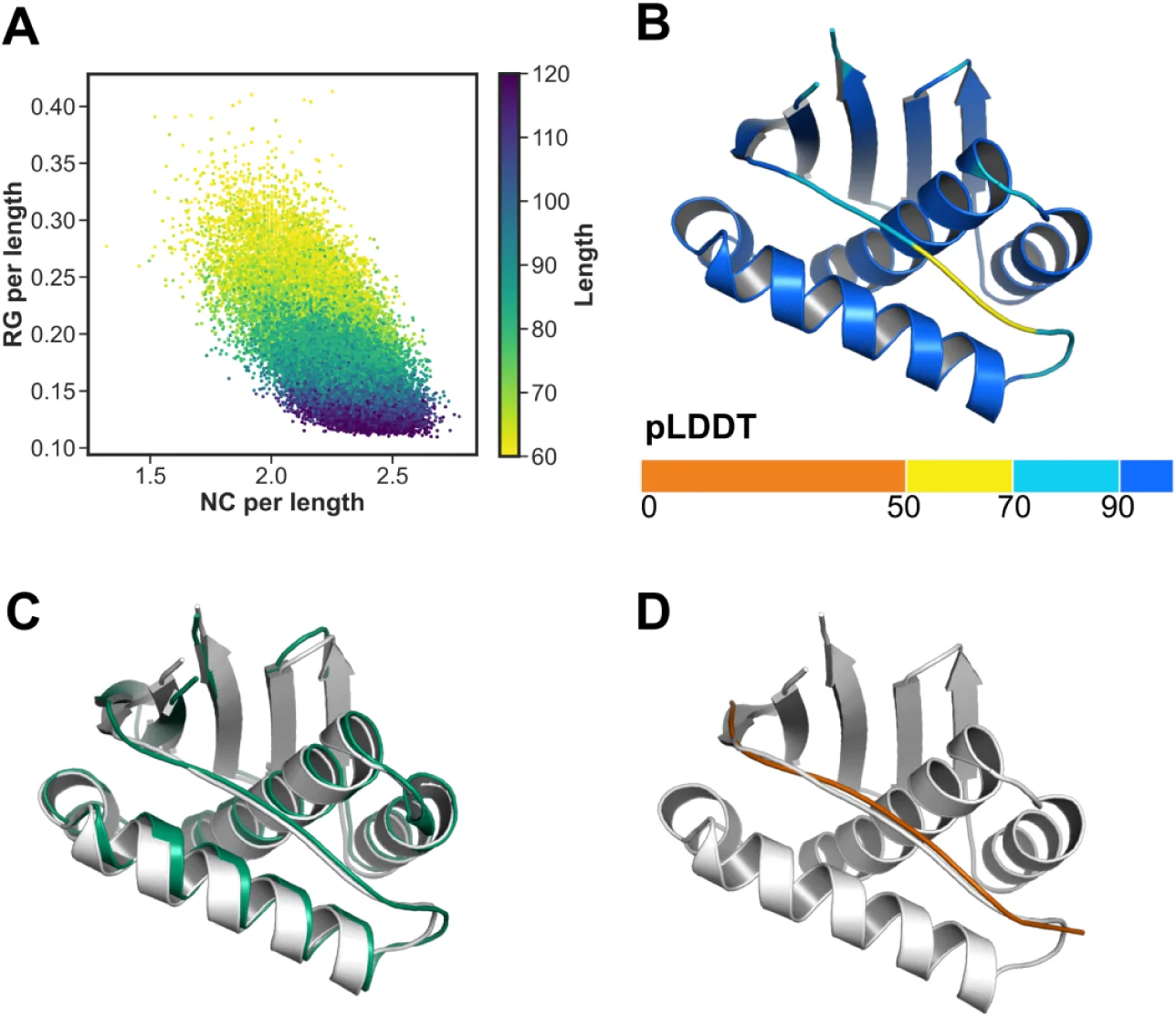
Top-ranked designed protein containing the selected motif from the YFV NS1 protein. (a) Scatter plot showing the distribution of radius of gyration (RG) versus number of backbone contacts (NC), both normalized by protein length, for each backbone generated by RFdiffusion. (b) Predicted structure of the designed protein colored according to the AlphaFold 3 pLDDT confidence score. (c) Superimposition of the designed protein (white) and its RFdiffusion-generated backbone (green). (d) Superimposition of the predicted protein structure and its native epitope (orange).

After reverse folding and full-atom structure prediction, the protein exhibiting the lowest Root Mean Square Deviation (RMSD) to the native peptide and the highest pTM score was selected (Figure 2B). Although predicting the structure of only a small subset of the generated backbone space may overlook many potentially designable scaffolds, this limitation did not affect our analysis, as the objective was to identify a single successful design for *in vitro* validation. The predicted tertiary structures exhibited low RMSD values relative to the native epitope (RMSD_peptide_), with 50% of designs displaying values below 4.82 Å, as well as low RMSD values relative to their corresponding generated backbones (RMSD_backbone_), with 38% of designs exhibiting values below 2.00 Å. These results compare favorably with previously reported *de novo* protein designs, for which RMSD backbone values below 2.00 Å are generally considered indicative of successful folding.^41^ Among these, the top-ranked candidate, hereafter referred to as YFV-Scaffold, exhibited an RMSD_backbone_ of 1.07 Å and an RMSD_peptide_ of 3.61 Å.

Additionally, BLASTp searches for sequences similar to YFV-Scaffold did not identify any relevant matches, indicating that the designed sequence has no detectable homologs in currently available databases. Likewise, structural similarity searches using FoldSeek revealed only limited and likely nonspecific matches (Table S1), with low probabilities and weak structural correspondence to proteins with unrelated biological functions from distant species, such as archaeal reductases. These findings suggest that YFV-Scaffold adopts a previously unreported protein architecture designed for its intended diagnostic purpose. This combination of sequence and structural novelty is desirable for diagnostic applications, as it minimizes the risk of off-target interactions with preexisting antibodies in patient sera.^16^ Unlike designs based on natural protein scaffolds, which may share epitopes with common human pathogens or self-antigens, our *de novo* scaffold is non-native to the human and microbial proteome, potentially reducing background serological reactivity.^42^

YFV-Scaffold was further characterized *in silico*, with the results supporting the success of the *de novo* design. Specifically, the epitope exhibited comparable levels of solvent exposure and a similar number of stabilizing contacts in both the designed scaffold and its native structural context (Figure 3A–B). Preservation of solvent accessibility is critical for diagnostic function, as buried epitopes are less accessible to antibody recognition.^35^ The similar contact profiles suggest that the designed scaffold successfully recapitulates the native epitope microenvironment, a challenge that has historically limited the success of epitope-scaffolding approaches.^43^ Likewise, the electrostatic surface potential of the peptide was preserved, with many residues in both the N- and C-terminal regions retaining similar charge distributions and exhibiting only minor differences in side-chain conformations, despite the distinct molecular environments of the native protein and the scaffolded region (Figure 3C).

**Figure 3:**
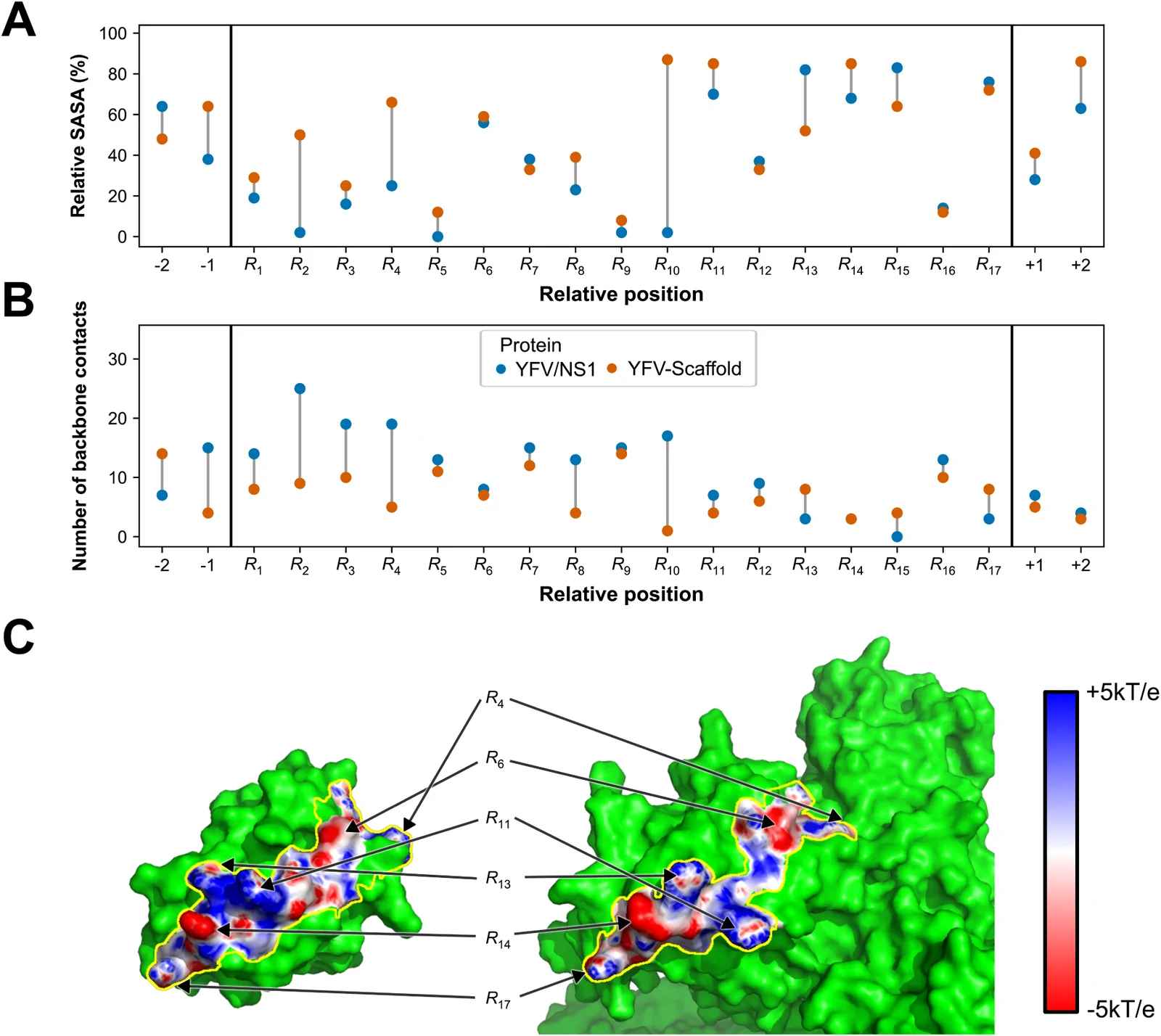
Comparison of the structural properties of the epitope in its native conformation and within the designed YFV NS1 protein. (a) Relative solvent-accessible surface area (SASA) per residue for the native epitope (blue) and the designed protein (orange). (b) Number of backbone contacts per residue, defined as the number of atoms from adjacent residues within 4 Å, using the same color scheme as in (a). (c) Electrostatic potential surface of the epitope in the native (right) and designed (left) protein contexts. Electrostatic potential ranges from −5 kT/e (red) to +5 kT/e (blue) and is mapped onto the van der Waals surface.

Additionally, the protein exhibited high predicted solubility, with a normalized score of 0.923 (i.e., higher than 92% of *Escherichia coli* proteins), according to Protein-Sol estimates. Consistent with this prediction, YFV-Scaffold reached high expression levels in *E. coli*, with no detectable aggregation and a purity of 95.3% (Figure 4A); in contrast, computational protein design protocols often yield multiple insoluble candidates.^44^ Circular dichroism spectra further revealed remarkable preservation of secondary structure across a broad temperature range, with minimal changes observed between 22 and 94 °C, followed by recovery of the original signal upon cooling, indicating high thermostability and reversible refolding (Figure 4B). This thermostability exceeds that of most natural viral proteins, which typically denature irreversibly above 60–70 °C^45^ The refolding capability is particularly valuable for diagnostic applications in resource-limited settings, where cold-chain storage may be unreliable.^46^ Together, these results demonstrate the effectiveness of the proposed design strategy in producing stable and potentially functional proteins.

**Figure 4:**
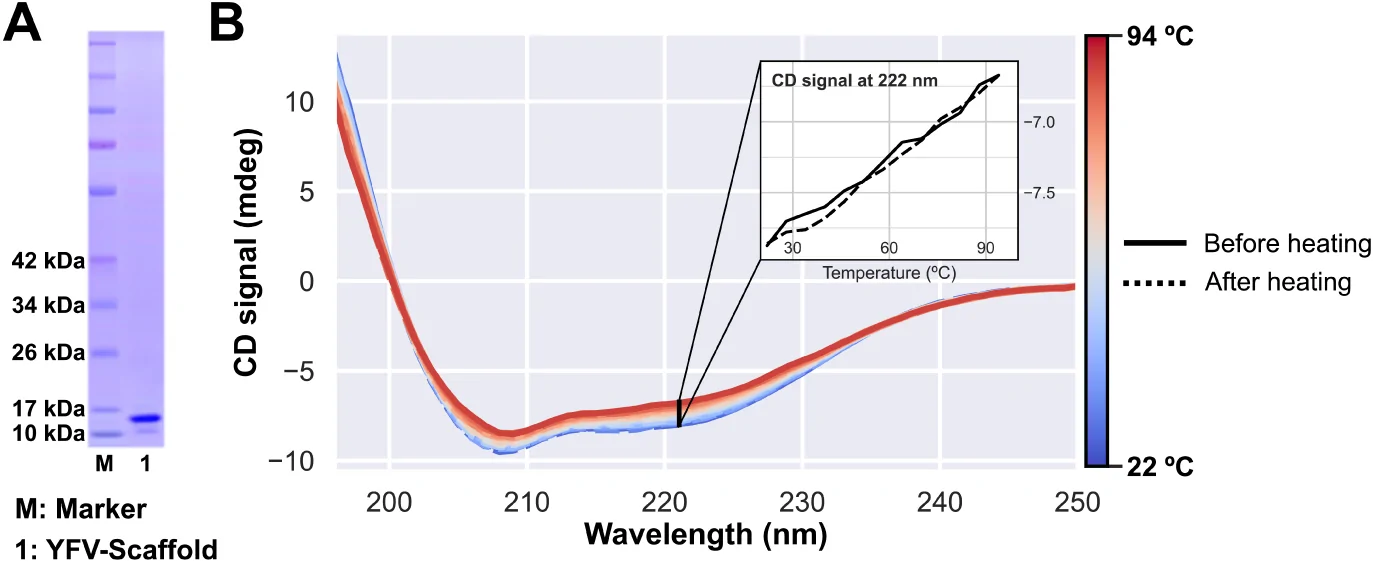
*In vitro* characterization of YFV-Scaffold. (a) Coomassie-stained SDS-PAGE gel confirming the purity of YFV-Scaffold. Lane M corresponds to the molecular weight marker, ranging from 10 to 42 kDa, and lane 1 contains YFV-Scaffold at approximately 15 kDa. (b) Circular dichroism spectra of the protein before and after heating. Ellipticity was measured from 196 to 250 nm at 6 °C intervals during heating from 22 °C to 94 °C (solid lines) and subsequent cooling back to 22 °C (dashed lines). Inset chart indicates the change in ellipticity at 222 nm as a function of the temperature.

### *In Vitro* Validation

#### Protein and Peptide Experimental Evaluation

YFV-Scaffold was compared with NS1 proteins from YFV, DENV, and ZIKV, as well as with the purified peptide, to assess its reactivity against anti-DENV and anti-YFV NS1 IgG antibodies (Figure 5A–B). Similar to YFV NS1, YFV-Scaffold exhibited high reactivity against polyclonal anti-YFV NS1 antibodies, whereas the free peptide showed limited or no reactivity, indicating that the surrounding protein structure plays a critical role in preserving the native conformation of the epitope and its antibody-binding properties. These findings align with a growing consensus in epitope-based diagnostics that linear peptides often fail to recapitulate conformational epitopes, resulting in reduced sensitivity.^47^ The successful restoration of binding activity through scaffolding demonstrates the potential of *de novo* protein design to stabilize flexible peptide motifs in bioactive conformations, an approach that has gained traction in vaccine design^16^ but remains underexplored for diagnostic applications.

**Figure 5:**
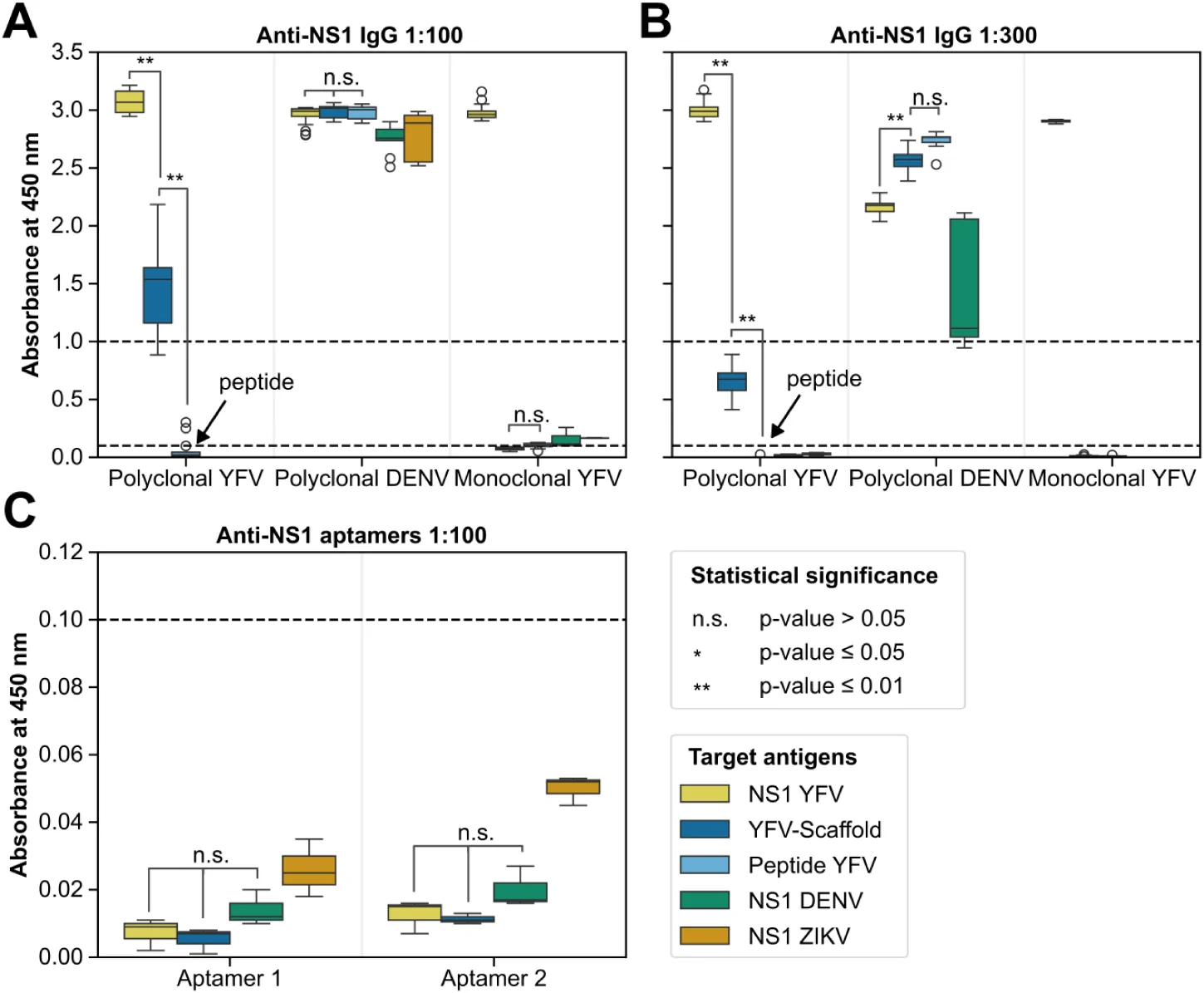
Indirect ELISA absorbance readings obtained for each antibody or aptamer against the indicated antigen coatings. Dotted lines denote the thresholds used to classify results as negative (<0.1), inconclusive (0.1–1.0), or positive (>1.0). Asterisks indicate the level of statistical significance (*p ≤ 0.05, **p ≤ 0.01), whereas non-significant differences are indicated by n.s. (a) Absorbance readings for antibodies at a 1:100 dilution. (b) Absorbance readings for antibodies at a 1:300 dilution. (c) Absorbance readings for aptamers at a 1:100 dilution.

Beyond demonstrating the importance of structural scaffolding for antibody recognition, we next evaluated whether YFV-Scaffold was similarly recognized by distinct antibody repertoires. Both YFV-Scaffold and the peptide exhibited low reactivity against monoclonal anti-YFV antibodies, indicating that recognition by this antibody likely requires a specific epitope that is absent in both systems. The differential reactivity observed between polyclonal and monoclonal antibodies is expected, as polyclonal sera contain a diverse repertoire of antibodies recognizing multiple epitopes on NS1, whereas monoclonal antibodies target a single, often conformation-dependent epitope.^48^ Furthermore, all proteins, as well as the peptide, showed high reactivity against anti-DENV polyclonal antibodies, demonstrating substantial cross-reactivity and a limited ability to distinguish between DENV, YFV, and ZIKV infections.^8^ This phenomenon is a well-recognized challenge in flavivirus serology, driven by the high amino acid similarity among NS1 proteins (approximately 50–60% between DENV and YFV) and immune imprinting, whereby prior exposure to one flavivirus may bias subsequent antibody responses toward cross-reactive epitopes.^8^

Importantly, our design did not aim to eliminate cross-reactivity at the level of a single epitope; rather, it sought to reproduce the reactivity profile of full-length NS1 using a simpler and more manufacturable protein. The comparable reactivity of YFV-Scaffold and full-length NS1 against polyclonal anti-YFV antibodies suggests that the scaffold captures a substantial fraction of the antibody repertoire directed against NS1. These results indicate that YFV-Scaffold represents a viable alternative to full-length YFV NS1 for diagnostic applications, exhibiting a comparable reactivity profile against surrogate antibodies while offering the advantages of a smaller and less complex structure and the absence of glycosylation sites, features that may facilitate large-scale production and reduce manufacturing costs.

To our knowledge, this study provides the first demonstration of a *de novo* designed protein capable of reproducing the serological reactivity of a full-length viral antigen for diagnostic purposes. Previous studies have focused on epitope-scaffolded immunogens for vaccination^49^ or on computationally redesigned natural proteins for diagnostic applications.^50^ Our approach extends these principles to the *de novo* design of diagnostic reagents, providing a potential route to replace complex and costly natural antigens with synthetic, readily producible alternatives.

#### Aptamers Experimental Evaluation

In parallel with the *de novo* protein engineering strategy, we explored an alternative molecular recognition approach based on the *in silico* design of aptamers targeting the selected YFV NS1 epitope. The resulting sequences were synthesized and experimentally evaluated alongside anti-YFV NS1 IgG antibodies. However, none of the designed aptamers exhibited detectable binding to the target proteins (Figure 5C), as evidenced by no positive or borderline absorbance values observed. Several factors may account for this lack of reactivity. Some limitations are inherent to aptamer development, including suboptimal sequence length, insufficient structural stability, or inadequate affinity for short peptide targets.^51^ Aptamers typically require targets with sufficient accessible surface area to establish high-affinity interactions,^52^ and short linear peptides may not provide an adequate binding interface. In addition, challenges specific to computational aptamer design may have contributed to the observed results. In particular, the predicted secondary and tertiary structures may not have been stably adopted under the experimental conditions, potentially reflecting limitations in the size, diversity, or representativeness of the datasets used to train current prediction models.^22^ Unlike conventional SELEX, which typically selects aptamers against full-length proteins or other complete molecular targets, our computational approach sought to generate aptamers directed toward a specific NS1 peptide epitope. Although this strategy has the potential to increase target specificity, it also imposes additional constraints on molecular recognition, as short peptides provide a more limited interaction surface than complete proteins. Furthermore, purely computational approaches do not fully account for solution dynamics, ionic effects, or the conformational flexibility of RNA molecules.^21^ Taken together, these findings highlight the current limitations of purely *in silico* aptamer design and underscore the continued need for experimental validation and iterative optimization in developing diagnostic binders through computational approaches.

## Conclusions

Our study introduces a novel approach that integrates evolutionary genomics with *de novo* protein design, providing a generalizable framework for the development of cost-effective serological diagnostic antigens for neglected arboviruses. Using this approach, we designed YFV-Scaffold, a *de novo* protein incorporating a temporally conserved region of the YFV NS1 protein. In an in-house ELISA assay, YFV-Scaffold exhibited a reactivity profile comparable to that of commercially available full-length YFV NS1 when tested against anti-YFV and anti-DENV polyclonal antibodies.

We further demonstrate that evolutionary information can be leveraged not only to identify conserved antigenic regions but also to guide the rational design of simplified protein mimics with structural and functional properties comparable to those of native viral antigens. YFV-Scaffold preserved key features of the native epitope, including solvent accessibility, local structural environment, and electrostatic properties, while exhibiting high solubility, thermostability, and recombinant expression levels. In contrast, the isolated peptide failed to reproduce the antibody reactivity observed for either full-length NS1 or YFV-Scaffold, highlighting the critical role of structural scaffolding in maintaining epitope recognition.

Conversely, the lack of detectable binding by the designed aptamers underscores the current challenges associated with purely *in silico* binder development, particularly when targeting short peptide epitopes. Although flaviviral cross-reactivity remains an important limitation and validation using human patient sera is still required, YFV-Scaffold represents a promising alternative to full-length NS1 for diagnostic applications. More broadly, the modular nature of this framework enables its adaptation to other pathogens, providing a scalable route for the development of stable, low-cost, and readily manufacturable diagnostic reagents.

## Materials and Methods

To identify suitable peptides for protein design, we initially collected all available high-quality YFV, DENV-1–4, and ZIKV genome sequences representative of the known viral diversity and performed evolutionary analyses. Candidate regions were then filtered using structural criteria, including surface accessibility and physicochemical properties. Protein design proceeded through backbone generation and protein-features filtering, followed by sequence design. Each designed protein was then scored based on structure prediction accuracy and similarity to the native epitope, and the best candidate was selected for validation. The following sections outline the workflow in detail.

### Sequence Acquisition

YFV, DENV-1–4 and ZIKV genomic sequences and their sampling dates were retrieved from the Bacterial and Viral Bioinformatics Resource Center (BV-BRC) database (https://www.bv-brc.org/) (Table 1). Inclusion criteria comprised complete genomic sequences of distinct lineages and genotypes of the flaviviruses YFV, DENV-1–4, and ZIKV obtained from human hosts, spanning different sampling years. Duplicated and low-quality sequences containing degenerate nucleotides were removed using Sequence Cleaner (https://github.com/metageni/Sequence-Cleaner). A complete list of BV-BRC and GenBank accession numbers, isolate names, geographical locations, and collection dates for all sequences used in this analysis is provided in Table S2. Subsequently, multiple sequence alignment (MSA) was performed using MAFFT v7.505 ^53^ and manually edited in AliView v1.30 ^54^ to ensure codon alignment.

**Table 1:** Summary of the Sequence Dataset Used in This Study

Virus	No. of representative sequences	No. of downloaded sequences	Collection dates
YFV	120	210	1927–2023
ZIKV	448	729	2006–2021
DENV-1	2310	2664	1944–2021
DENV-2	1537	1734	1944–2021
DENV-3	1125	1284	1956–2022
DENV-4	422	565	1956–2021

### Bayesian Phylodynamics

Given the limited number of available YFV sequences, nucleotide substitution models were selected using the Akaike Information Criterion corrected for small sample sizes (AICc) to minimize the risk of model overfitting, as implemented in ModelTest-NG v0.2.0.^55^ The General Time Reversible model with gamma-distributed rate variation among sites and a proportion of invariant sites (GTR + Γ4 + I) was selected as the best-fitting model. Temporal signal assessment and preliminary estimation of the time to the most recent common ancestor (tMRCA) were performed using TempEst v1.5.3,^56^ based on maximum likelihood phylogenies inferred with RAxML v8.2.12 ^57^ under the selected substitution model.

Phylodynamic trees were inferred using BEAST v1.10 package^58^ to reconstruct ancestral states and characterize temporal and evolutionary population dynamics. A lognormal relaxed molecular clock and a Bayesian SkyGrid coalescent prior^59^ were imposed and configured in BEAUti (https://beast.community/beauti), with the latter parameterized using one grid interval per year extending back to the estimated tMRCA. Markov chain Monte Carlo (MCMC) analyses were conducted in two independent runs, each of up to 500 million steps or until convergence was achieved, as indicated by effective sample size (ESS) values greater than 200 for all estimated parameters. Maximum clade credibility (MCC) trees were generated using TreeAnnotator v1.10.4 (https://beast.community/treeannotator) after discarding a 10% burn-in and combining two independent runs. Root age estimation was performed using Tracer v1.7.3 ^60^ to determine the tMRCA with 95% highest posterior density intervals.

Additionally, an in-house Python script (constant_in_time.py) was developed to identify genomic regions that remained stable over time. This script parses the posterior probability associated with each sequence and node of the MCC tree and computes the weighted consensus for the alignment at each node. Sites were classified as temporally stable if they exhibited posterior probabilities ≥99% across all nodes. Based on this criterion, the script generated a “temporal consensus” sequence in which only sites meeting this threshold were retained, while variable positions were masked. Tree visualization and figure generation were performed using TreeViewer v2.2.0.^61^

### Selection and Recombination Analyses

The presence of codons under diversifying selection was evaluated using multiple selection-detection methods implemented in the DataMonkey web server v2 (https://www.datamonkey.org/).^62^ Statistical tests included the Mixed Effects Model of Evolution (MEME) for episodic selection;^30^ Fixed Effects Likelihood (FEL) and Single-Likelihood Ancestor Counting (SLAC) for pervasive selection;^63^ and Fast Unconstrained Bayesian Approximation (FUBAR).^64^ Codons were considered under positive selection if they met predefined significance thresholds: p-value ≤ 0.05 (MEME, FEL and SLAC) or posterior probability ≥ 0.9 (FUBAR). To assess potential recombination events, the Genetic Algorithm for Recombination Detection (GARD) method was applied using a general discrete model with codon-position-aware partitioning.^65^

### Prediction of MHC-Binding and B-Cell Epitopes

To identify regions with the highest immunogenic potential and support epitope selection, binding predictions were performed for both MHC class I and class II molecules against the NS1 consensus sequence. Predictions for MHC class I binding were carried out using NetMHCpan v4.1b,^66^ screening all available linear peptides against HLA supertype representatives and the ten most frequent South and Central American alleles according to https://www.allelefrequencies.net (Table S3). Peptides ranking within the top 0.5% of predictions based on ligand likelihood scores were considered strong binders. MHC class II binding predictions were performed using NetMHCIIpan v4.3e,^66^ incorporating protein context-aware predictions to improve predictive accuracy.

Additionally, B-cell epitopes prediction was carried out with BepiPred v3.0 ^67^ against the NS1 consensus sequence. Per-residue epitope prediction scores were automatically converted into linear scores by BepiPred via sequential smoothing. Peptides were classified as epitopes only if all of their residues ranked within the top 10% of linear epitope prediction scores.

### Peptide Selection and Structural Analyses

In order to identify conserved motifs within the NS1 protein, we performed direct comparisons against representative flaviviruses of the same family, including DENV serotypes 1–4 and ZIKV, and used the evolutionary conservation profile generated by ConSurf.^68^ Each conserved motif was evaluated according to its isoelectric point and charge at pH 7.0 (calculated using Biopython v1.81),^69^ solvent-accessible surface area (SASA, calculated using PyMOL v3.0), and its proximity to glycan-shielded regions and known or predicted epitopes.

To calculate relative SASA, all available NS1 PDB structures for each virus were downloaded (see Table S4) and processed using PyMOL 3.0.^70^ In addition, AlphaFold3 ^71^ was used to predict the structure of the NS1 homotetramer, reported to be the predominant secreted oligomeric form of NS1 when compared with its dimeric and hexameric states.^72^ The impact of glycosylation on protein surface accessibility was also evaluated by inferring N- and O-glycosylation sites using NetNGlyc v1.0 (https://services.healthtech.dtu.dk/services/NetNGlyc-1.0/) and NetOGlyc v4.0,^73^ respectively.

Following glycosylation-site prediction and validation against available literature, glycans were grafted onto protein structures using the GlycoSHIELD web application^35^ to simulate glycan shielding and estimate surface occlusion levels. Glycans were selected based on their apparent size and complexity, with GlcNAc(2)­Man(9)­Glc(3) representing the high-mannose glycan type.

### *In Silico* Protein Design

Promising peptides identified through the analyses described above were incorporated into scaffolded proteins designed *de novo* using the motif-scaffolding method implemented in RosettaFold diffusion (RFdiffusion) v1.1.0 commit #820bfdf.^37^ A total of 80,000 protein backbones were generated, with lengths ranging from 60 to 120 residues, and ranked by radius of gyration (Rg) and number of contacts (NC), both normalized by protein length. Backbones with no more than 0.1 Å per residue from the best normalized Rg value across all generated structures and 0.15 Cα pairs per residue from the best normalized NC value across all generated structures were selected for further analysis. The secondary structure of each residue in the backbone structures was assigned using a simplified implementation of the DSSP algorithm (https://pypi.org/project/pydssp/),^74^ and the proportion of α-helices was used as a proxy for structural diversity among the generated backbones. For each selected protein backbone, 250 amino acid sequences were designed using ProteinMPNN.^75^ The sequence with the lowest global score was selected for tertiary structure prediction using AlphaFold3 and subsequently evaluated based on its pTM score, RMSD relative to the corresponding generated backbone (RMSD_backbone_), and RMSD between the designed motif and its PDB counterpart (RMSD_motif_).

The generated protein structures were also characterized using FoldSeek,^76^ enabling structural comparisons against known protein databases to identify structurally similar proteins. Additionally, to further validate these results and minimize the risk of false positives, sequence-based homology searches were conducted using BLASTp. The predicted solubility of each protein was estimated using the Protein-Sol web server^77^ to assess the risk of aggregation during recombinant production. Electrostatic potentials for both native NS1 and YFV-scaffold were calculated using the linearized Poisson–Boltzmann equation using the APBS software^78^ with the solvent at a saline (monovalent ions with 2 Å radius each) concentration of 0.15M. PDB 2PQR^79^ was used to generate the PQR formatted files from the PDB coordinates by assigning partial atomic charges for the proteins using the PARSE force field at pH 7.0. Structural visualization and molecular interaction analysis were carried out in PyMOL v3.0, allowing inspection of key residues, secondary structure elements, and physicochemical properties. Finally, the protein structure with the lowest RMSD and highest pTM score was selected for further *in vitro* analyses.

### *In Silico* Aptamer Design

To generate aptamer candidates, 50 RNA sequences were generated against each target region of the YFV NS1 protein using APTA-MCTS,^80^ employing a classifier trained on the dataset described by Li et al.^81^ The classifier used the target motif as a positive criterion, while the remainder of the viral protein and the corresponding region in other flaviviruses were used as negative criteria to reduce the likelihood of cross-reactivity. The tertiary structures of the generated aptamers were then predicted using SimRNA,^82^ RNAComposer,^83^ and AlphaFold3. We then performed docking simulations using ZDOCK3 ^84^ to assess the ability of the predicted aptamers to bind the target protein motif. The two sequences with the highest ZDOCK3 scores were selected for further analysis.

### Production of YFV-Scaffold, Purified Peptide, and Aptamers

YFV-Scaffold protein was synthesized by LifeTein LLC (New Jersey, USA) via expression in *E. coli* Top10 strain using a pET-28a­(+) cloning vector. The protein was purified (≥90%) by nickel affinity chromatography, and purity was confirmed by SDS-PAGE, followed by lyophilization. To determine whether reactivity required preservation of the peptide’s native three-dimensional conformation within the designed protein or could be achieved by the peptide alone, we also tested the isolated peptide *in vitro*. The purified peptide (95.3%) was produced by GenScript (New Jersey, USA) via PepPower chemical synthesis, which included purification by high-performance liquid chromatography (HPLC). The peptide sequence was confirmed by mass spectrometry. The aptamers were synthesized by GenScript (Hong Kong, China) and labeled with a 5′-Biotin-TEG moiety for experimental use, as well as with three 2′-O-methyl and phosphorothioate modifications at each terminus to increase stability. Aptamer purity (≥99%) was confirmed by the company using RNase-free HPLC.

### Circular Dichroism

Circular dichroism (CD) experiments were conducted using a Chirascan Circular Dichroism Spectropolarimeter (Applied Photophysics, UK) with a quartz cuvette of 0.1 cm path length and a 3 nm bandwidth. Far-UV spectra (196–250 nm) were obtained from three scans acquired at 60 nm/min and averaged, and buffer baselines were subtracted from the corresponding sample spectra. Protein unfolding and refolding were monitored over a temperature range of 22–94 °C in 6 °C increments, with a 3 min equilibration period at each temperature before spectra were recorded during both heating and cooling cycles.

### In-House Indirect ELISA

First, mouse monoclonal anti-YFV NS1 IgG (Merck Millipore, Darmstadt, Germany), rabbit polyclonal anti-YFV NS1 IgG (Merck Millipore, Darmstadt, Germany), and human polyclonal anti-DENV NS1 IgG (EUROIMMUN, São Paulo, Brazil) were biotinylated using the EZ-Link Sulfo-NHS-LC-Biotinylation Kit (Thermo Fisher, Illinois, USA) according to the manufacturer’s instructions. The antibodies were serially diluted and applied to Nunc MicroWell 96-well microplates (Nunc, New York, USA) to determine the antibody concentrations that produced absorbance values comparable to those observed in the anti-DENV NS1 IgG ELISA assay from which the antibodies were obtained.

Then, 96 well microplates were coated with 100 μL per well of purified ZIKV NS1 (The Native Antigen Company, Kidlington, UK), recombinant YFV NS1 (R&D Systems, Minneapolis, USA), or YFV-Scaffold at 4 μg/mL and incubated overnight at 4 °C. Subsequently, microplates were washed with phosphate-buffered saline (PBS) containing 0.05% Tween-20 and blocked for 2 h at room temperature with 200 μL of blocking buffer (a 1:1 mixture of 1% bovine serum albumin (BSA) and 3% skim milk powder in PBS). Optimized concentrations of biotin-labeled aptamers and anti-YFV NS1 and anti-DENV NS1 antibodies were added in triplicate (100 μL per well) and incubated for 1 h at 37 °C. After washing, 100 μL of streptavidin-conjugated horseradish peroxidase (HRP) was added to each well, and the plate was incubated for 30 min at room temperature. Following another wash, 100 μL of 3,3″,5,5″-tetramethylbenzidine (TMB) substrate solution was added to each well, and the reaction was stopped after 15 min by adding 100 μL of 0.5 M sulfuric acid. Absorbances were measured at 450 nm using an LMR-96 Microplate Spectrophotometer (Loccus, São Paulo, Brazil). The cutoff value was defined as the mean optical density (OD) of the negative controls. Index values were calculated as the OD of each sample minus the cutoff value. Results above 1.0 were considered positive; those below 0.1 were considered negative; and values between 0.1 and 1.0 were classified as inconclusive. Positivity was calculated as the proportion of positive results among all true-positive YFV samples. Sensitivity was defined as the ratio of true positives to the sum of true positives and false negatives, excluding inconclusive results. Specificity was defined as the ratio of true negatives to the sum of true negatives and false positives, also excluding inconclusive results.

### Statistical Analyses

A two-way analysis of variance (ANOVA) was used to evaluate the effects of protein target and analyte concentration, as well as their interaction, on absorbance levels. To identify significant differences between specific groups, Tukey’s honestly significant difference (HSD) post hoc test was performed. A p-value ≤ 0.05 was considered statistically significant. All analyses were conducted using R statistical software version 4.5.3 (https://www.r-project.org/).

## Supplementary Material







## Data Availability

The Python scripts used in this study are available at https://github.com/jrom99/utilities/. Additional data supporting the findings of this study are available from the corresponding author upon reasonable request.

## Notes

Figure S1. Unique regions mapped onto the YFV NS1 tetramer (PDB: 8ZBA); Figure S2. Evolutionary and structural properties of the native NS1 protein; Figure S3. Secondary structure composition of the generated backbones scaffolds; Figure S4. YFV-Scaffold structure; Figure S5. Structural superposition of YFV-Scaffold and the native YFV NS1 protein (PDF)
Scheme S1. YFV-Scaffold structure (PDB)
Table S1. FoldSeek results for the designed protein; Table S2. Complete list of the BV-BRC and GenBank accession numbers, isolate names, geographical location and collection date for all sequences used in the analysis; Table S3. Predicted MHC Class I and II binding epitopes identified using NetMHCpan and NetMHCIIpan; Table S4. PDB structures used for each virus (XLSX)
