# Supplementary material for: Evolutionary Dynamics-Aware De Novo Design of a Thermostable NS1-Derived Diagnostic Protein for Yellow Fever Virus
Source: ACS Omega. 2026 Sep 4;11(37):56323–35. doi: 10.1021/acsomega.6c07158 (PMC13613397; doi:10.1021/acsomega.6c07158)
Supplement: Supplementary file 1 [file ao6c07158_si_001.pdf]

Evolutionary dynamics-aware *de novo* design of a thermostable NS1-derived diagnostic protein for Yellow Fever virus

Junior Olimpio Martins[1], Patrícia da Silva Antunes[1], Rodrigo Lopes Sanz Duro[1], Robert Andreata-Santos[2,3], Clara Lacerda de Athayde[1,4], Gustavo Cabral-Miranda[5,6], Luiz Mário Ramos Janini[7], Franca Fraternali[8], Carlos Henrique Bezerra da Cruz[§8], Ricardo Durães-Carvalho*[§,1,9]

[1]Department of Morphology and Genetics, Federal University of São Paulo, São Paulo-SP, Brazil.

[2]Laboratory of Urban Rodent Virology, Institut Pasteur de São Paulo, São Paulo-SP, Brazil.

[3]Postgraduate Program in Microbiology and Immunology, Federal University of São Paulo, São Paulo-SP, Brazil.

[4]Anhembi Morumbi University, São Paulo-SP, Brazil.

[5]Laboratory of Genetics and Molecular Cardiology, Heart Institute, Clinical Hospital, Faculty of Medicine, University of São Paulo, São Paulo-SP, Brazil.

[6]Institute of Tropical Medicine, Faculty of Medicine, University of São Paulo, São Paulo-SP, Brazil.

[7]Department of Microbiology, Immunology and Parasitology, Federal University of São Paulo, São Paulo-SP, Brazil.

[8]University College London, Department of Structural and Molecular Biology, Darwin Building, 604 Gower Street, London, WC1E 6BT, United Kingdom.

[9]Interunit Bioinformatics Graduate Program, Institute of Chemistry, University of São Paulo, São Paulo-SP, Brazil.

*Correspondence: Ricardo Durães-Carvalho, UNIFESP Department of Morphology and Genetics, São Paulo - SP 04039-032, Brazil. E-mail: ricardo.duraes@unifesp.br

[§]Equal contribution as senior author.

Supporting information - Graphics

Figure S1

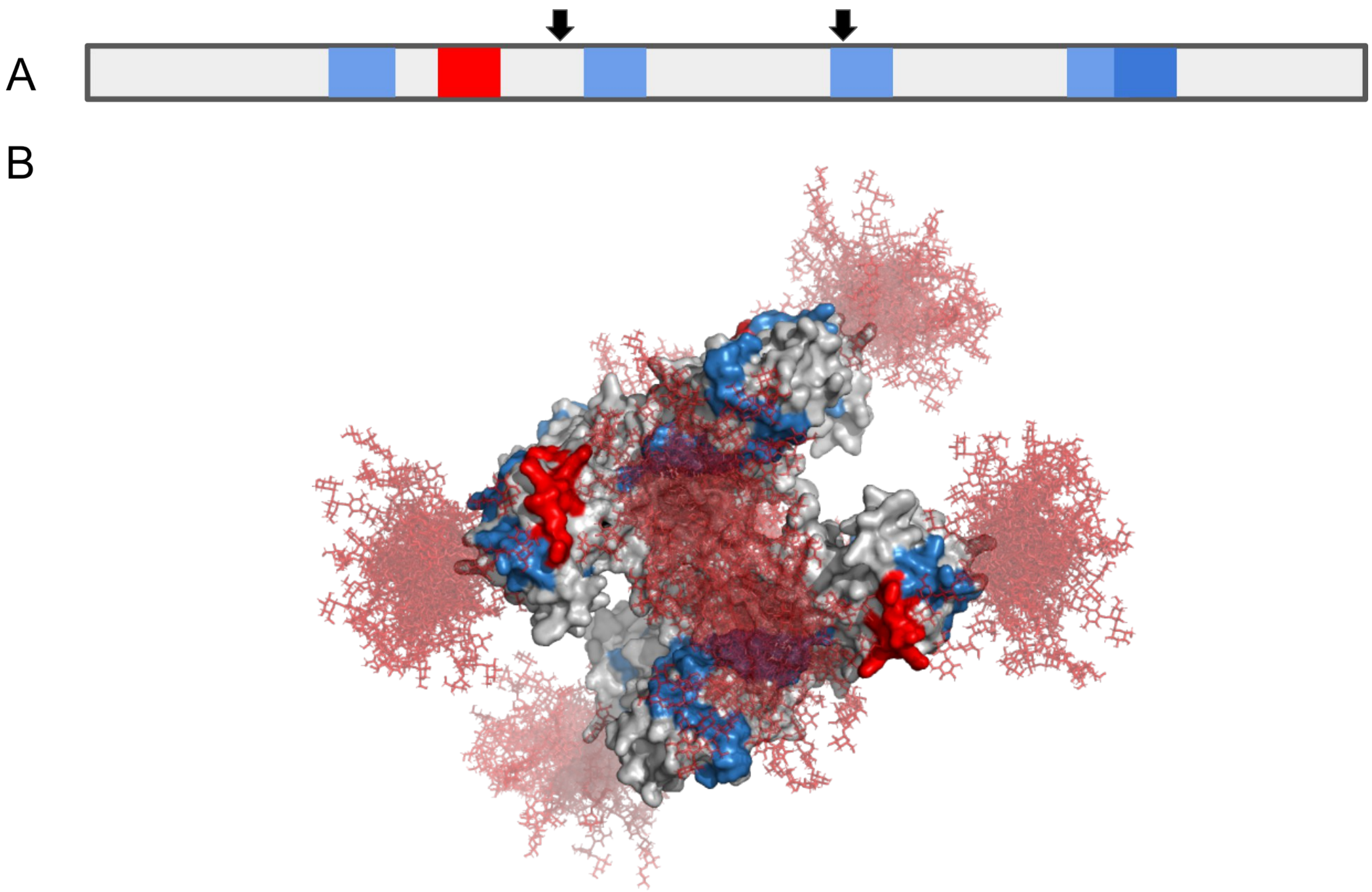

Figure S1. Unique regions mapped onto the YFV NS1 tetramer (PDB: 8ZBA). (a) Schematic representation of the NS1 protein sequence. The region selected for downstream analyses is highlighted in red, whereas the remaining mapped regions are shown in blue. Predicted glycosylation sites are indicated by arrows. (b) Glycan conformers generated using GlycoSHIELD are shown in light red.

Figure S2

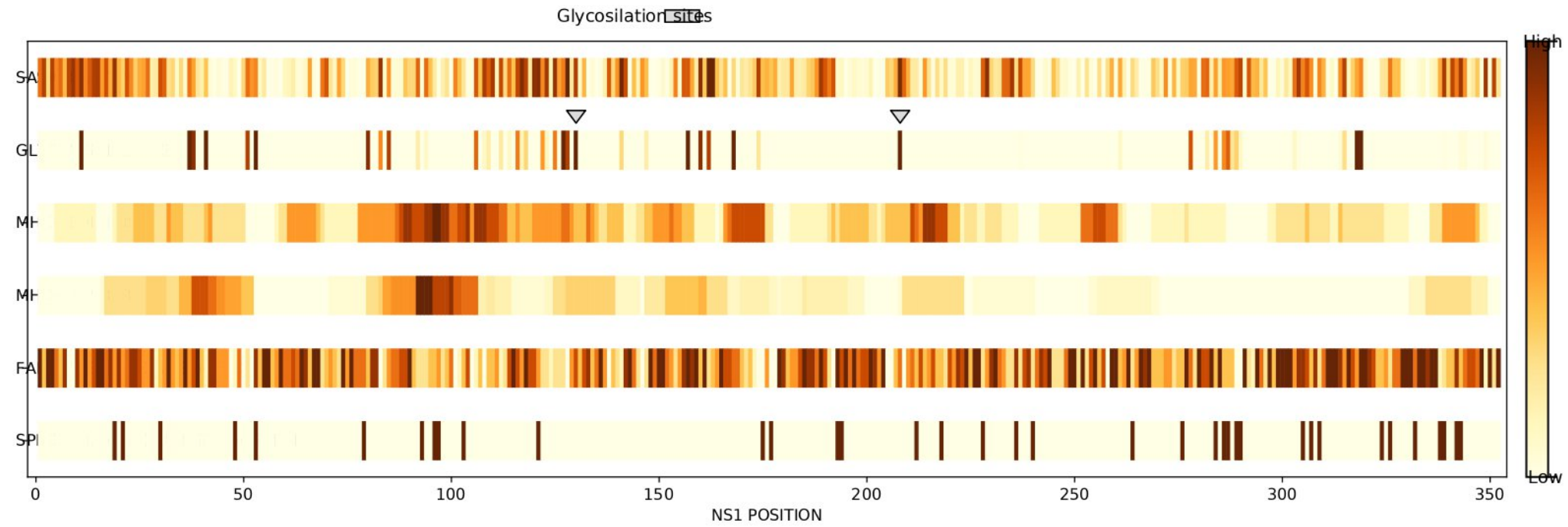

Figure S2. Evolutionary and structural properties of the native NS1 protein. Each row represents a different feature, including relative solvent-accessible surface area (SASA RELATIVE), steric occlusion by glycan conformers (GLYCAN SHIELDING), number of predicted major histocompatibility complex (MHC) class I binding epitopes (MHC-I BINDERS), number of predicted MHC class II binding epitopes (MHC-II BINDERS), evolutionary conservation inferred using ConSurf (FAMILY-LEVEL CONSERVATION), and amino acid substitutions inferred through ancestral character reconstruction across the YFV phylogeny (SPECIES-LEVEL CHANGES OVER TIME). Grey arrows indicate known glycosylation sites.

Figure S3

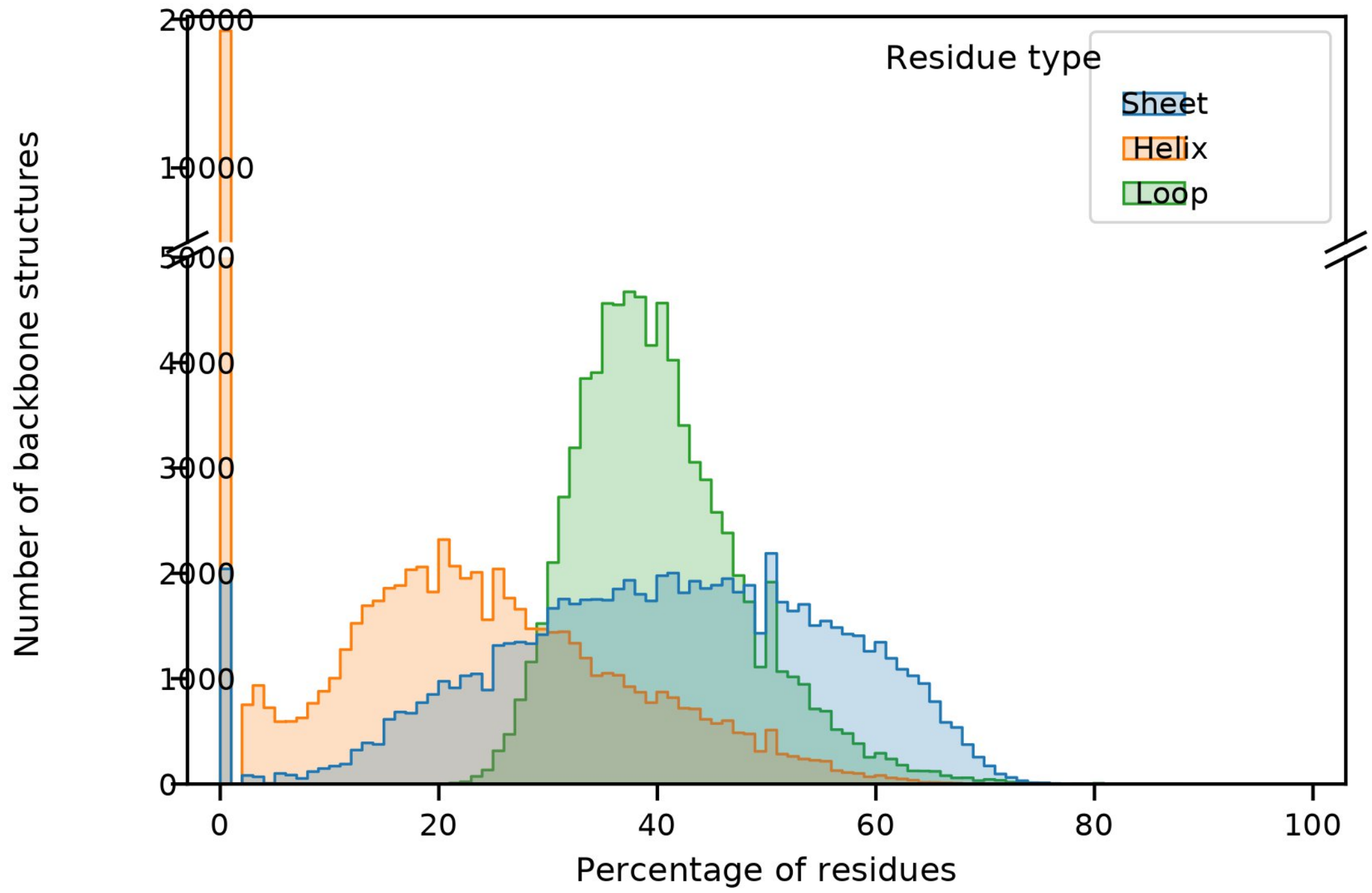

Figure S3. Secondary structure composition of the generated backbones scaffolds. Histogram showing the proportion of residues assigned as loop (green), α-helix (orange) or β-sheet (blue) secondary structure elements across all backbones containing the selected YFV NS1 peptide.

Figure S4

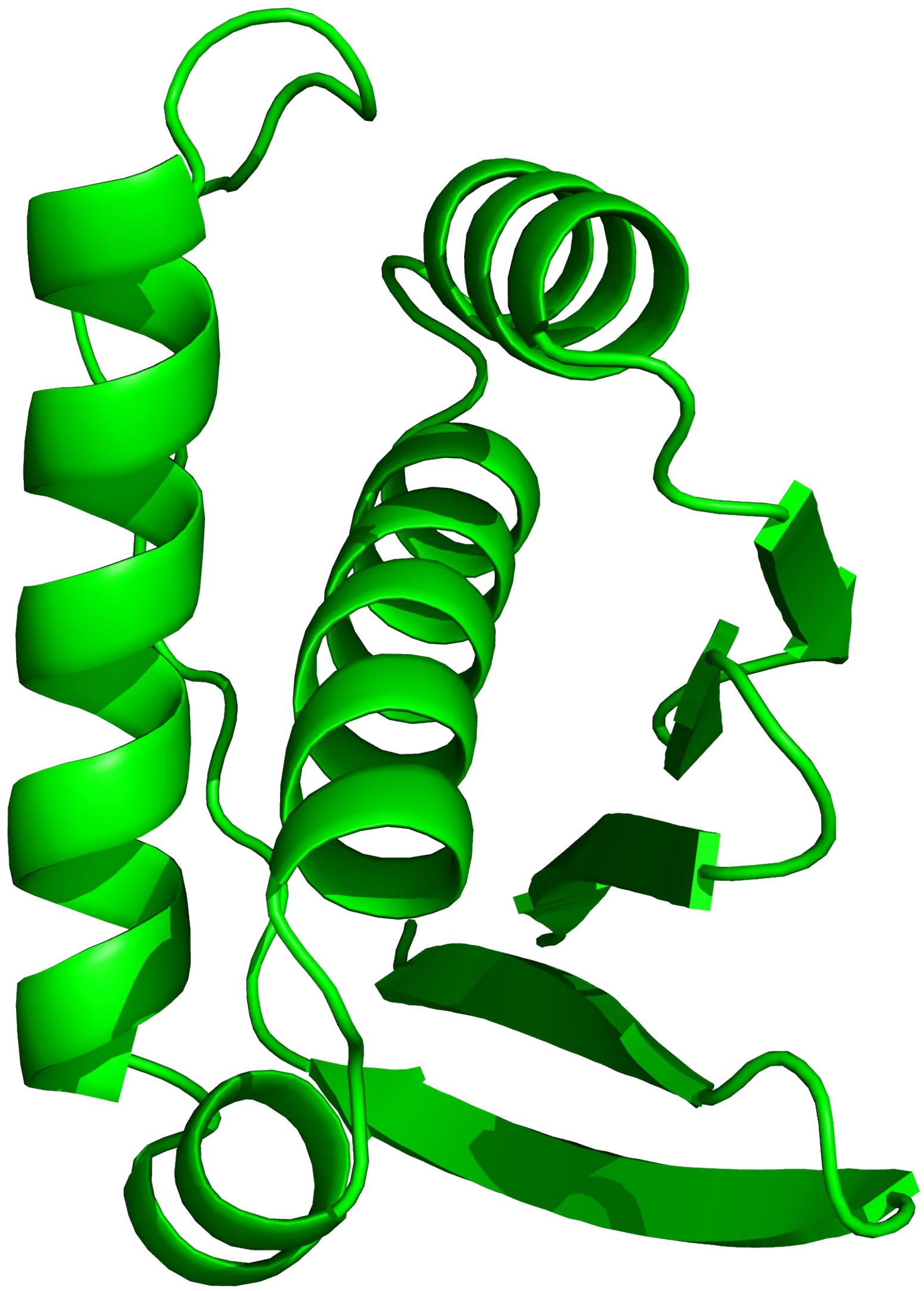

Figure S4. YFV-Scaffold structure.

Figure S5

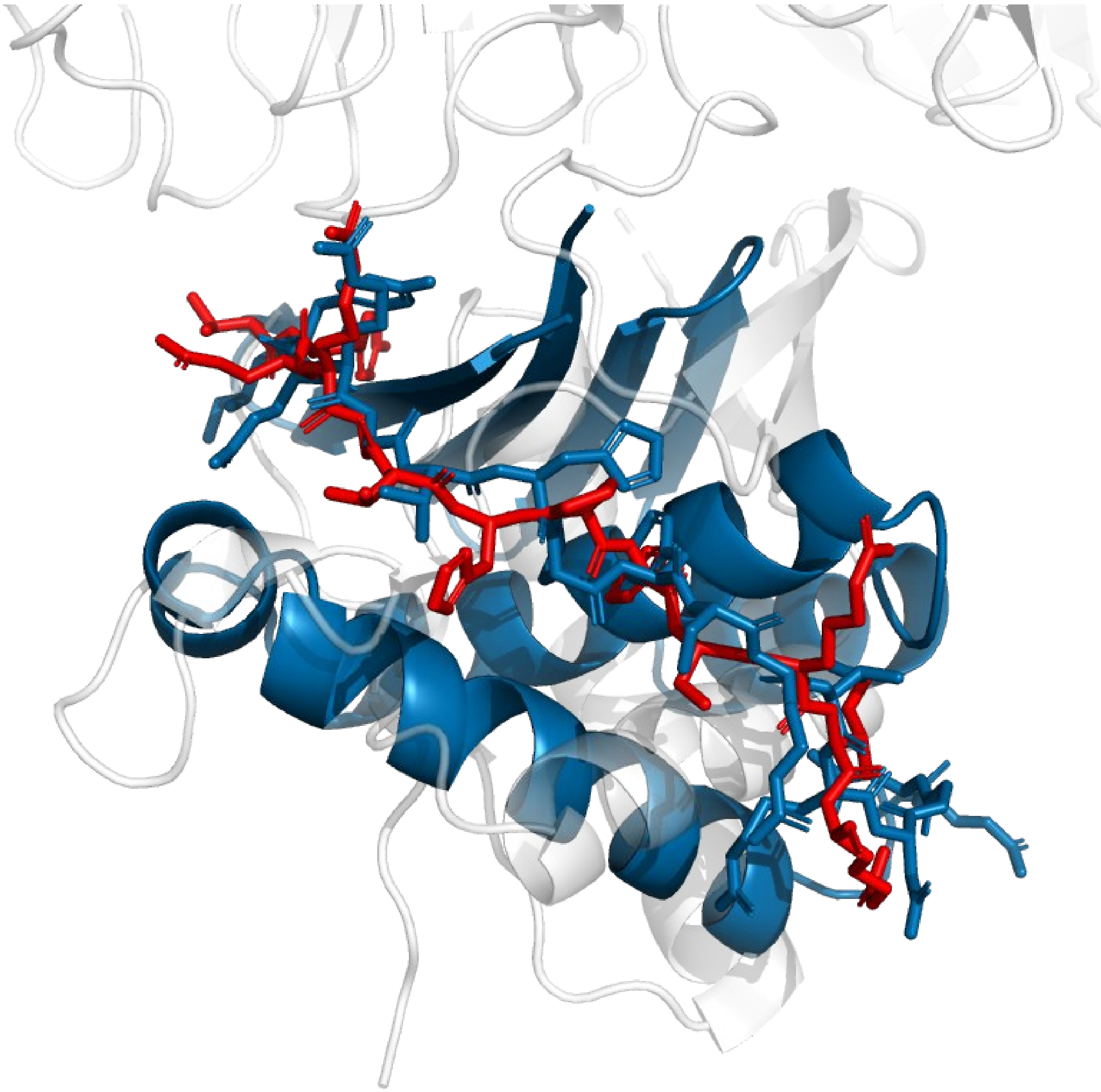

Figure S5. Structural superposition of YFV-Scaffold and the native YFV NS1 protein. The designed protein (YFV-Scaffold, blue) is superimposed onto the crystal structure of YFV NS1 (PDB ID: 8ZBA, white). Residues corresponding to the native epitope incorporated into the YFV-Scaffold design are highlighted in red.
